# In Vitro Evaluation of Drug–Drug Interaction Between Gliclazide and Antacids at the Absorption Level

**DOI:** 10.3390/ph18050684

**Published:** 2025-05-05

**Authors:** Slavica Lazarević, Srđan Kosijer, Maja Đanić, Dragana Zaklan, Bojan Stanimirov, Momir Mikov, Nebojša Pavlović

**Affiliations:** 1 Department of Pharmacology, Toxicology and Clinical Pharmacology, Faculty of Medicine, University of Novi Sad, 21000 Novi Sad, Serbia; slavica.lazarevic@mf.uns.ac.rs (S.L.); maja.djanic@mf.uns.ac.rs (M.Đ.); momir.mikov@mf.uns.ac.rs (M.M.); 2 Faculty of Medicine, University of Novi Sad, 21000 Novi Sad, Serbia; 014625@mf.uns.ac.rs; 3 Department of Pharmacy, Faculty of Medicine, University of Novi Sad, 21000 Novi Sad, Serbia; dragana.zaklan@mf.uns.ac.rs; 4 Department of Biochemistry, Faculty of Medicine, University of Novi Sad, 21000 Novi Sad, Serbia; bojan.stanimirov@mf.uns.ac.rs

**Keywords:** gliclazide, antacid, PAMPA, membrane, hydrotalcite, sulfonylurea, gastrointestinal

## Abstract

**Background**: The antidiabetic drug gliclazide is often taken with antacids due to its gastrointestinal side effects. However, patients rarely report antacid use, making drug–drug interactions a potential cause of therapy failure. Therefore, this study aimed to investigate the in vitro effects of various antacids on gliclazide permeability and to explore the underlying mechanisms. **Methods**: The permeability of gliclazide alone and in the presence of antacids (sodium bicarbonate, calcium carbonate, aluminum hydroxide, hydrotalcite and calcium carbonate/magnesium carbonate) was investigated using the parallel artificial membrane permeability assay (PAMPA) in four media (buffers pH 1.2, pH 4.5, pH 6.8 and water). The permeability coefficients were calculated, and the effect of pH on gliclazide permeability was also evaluated. **Results**: At simulated fasting gastric conditions (pH 1.2), groups with calcium carbonate, hydrotalcite and the combination of calcium carbonate/magnesium carbonate showed significantly higher permeability of gliclazide than the control group. At fed-state gastric conditions (pH 4.5), only hydrotalcite did not significantly change the permeability of gliclazide. Sodium bicarbonate, aluminum hydroxide and hydrotalcite significantly reduced the gliclazide permeability in comparison to the control group at pH 6.8 as a representative of fasted-state intestinal fluid. **Conclusions**: Antacids significantly impact the permeability of gliclazide at different pH values, potentially influencing its bioavailability. Gliclazide permeability is mainly influenced by pH-dependent ionization, though complex or salt formation may also play a role. Since both gliclazide and antacids are taken with food, and gliclazide is primarily absorbed in the small intestine, calcium- and magnesium-based antacids can be considered the most suitable choice.

## 1. Introduction

Gastroesophageal reflux disease (GERD) and heartburn represent prevalent clinical conditions among individuals diagnosed with type 2 diabetes mellitus. Different studies confirmed that upper gastrointestinal symptoms are more prevalent in patients with diabetes than in controls [1]. These symptoms are not unexpected given that hyperglycemia is associated with a disorder of gastric myoelectric activity and reduced motility in the stomach, duodenum and jejunum [2]. Furthermore, autonomic neuropathy and microangiopathy, which are frequent diabetic complications, may lead to gastric stasis and small bowel dysmotility [3]. Nevertheless, it may be challenging to distinguish between gastrointestinal symptoms, which are a consequence of the long-standing disease, and the side effects of the oral hypoglycemic drugs [4]. By any means, patients with diabetes frequently use gastric acid-reducing agents in an effort to alleviate these symptoms. Despite the availability of several therapeutic options, antacids are the mainstay treatment for upper gastrointestinal symptoms due to their safety, efficacy and over-the-counter (OTC) availability.

Antacids were traditionally regarded as the first-line choice for the treatment of symptoms associated with upper gastrointestinal tract diseases, such as peptic ulcer disease, gastroesophageal reflux disease, dyspepsia and others. With the introduction of more effective medications, such as H2 receptor antagonists and proton pump inhibitors, the use of antacids as first-line therapy for chronic acid-related disorders (e.g., GERD, peptic ulcer) has declined. However, their use remains prevalent due to their OTC availability [5]. Nonetheless, the practice of self-medication escalates the likelihood of clinically relevant interactions with other drugs that patients may be concurrently utilizing, particularly among those engaged in polypharmacy, defined as the concurrent use of five or more pharmacological agents [5,6].

Antacids are hydroxides or alkaline salts of magnesium, sodium, aluminum and calcium that exert their effects by directly neutralizing the effects of stomach acid. They are characterized by a rapid onset and short duration of suppression of gastric acid production. When taken 1 h after a meal, the effect usually diminishes after 2 h due to gastric emptying and acid secretion [5,7]. Previous studies have shown that interactions of antacids with specific drugs primarily affect the speed and extent of absorption of the simultaneously administered oral drug, which is the consequence of the acid-neutralizing capacity of antacids to change gastric pH, the formation of chelates and the alteration of the motility of the gastrointestinal tract [8,9].

Nevertheless, patients are usually unaware of these risks and do not consider antacids as drugs [5]. In cases of failure of antidiabetic therapy, drug–drug interactions (DDIs) should always be considered. However, patients generally do not inform physicians about the concomitant use of antacids.

A series of studies have been conducted to assess interactions between antacids and various sulfonylurea antidiabetic drugs in healthy volunteers and confirmed variations in absorption of these drugs [10,11,12]. The interactions between antacids and gliclazide, a drug classified within the second generation of sulfonylurea derivatives, remain insufficiently investigated. The variability in the bioavailability of gliclazide could affect a therapeutic response or lead to severe adverse effects such as hypoglycemia. Gliclazide is acknowledged as an effective oral therapeutic agent for managing type 2 diabetes mellitus as it achieves good glycemic control and diminishes markers of endothelial inflammation, thereby reducing the risk of serious microvascular and macrovascular complications [13,14]. It is usually prescribed if patients are intolerant to or do not respond to metformin monotherapy [15]. In terms of physicochemical properties, gliclazide is mainly classified as a weak acid drug with good lipophilicity (logP = 1.7) [13]. According to the Biopharmaceutical Classification System (BCS), gliclazide is classified into BCS class II based on its low solubility, which may result in its slow absorption in the gastrointestinal tract and susceptibility to interactions with other drugs in the resorption phase. The primary mechanism of gliclazide absorption is passive diffusion. It has been shown that pH values between 3.0 and 4.2 represent the range in which gliclazide absorption depends on solubility. When the pH value is higher than 4.2, the solubility of gliclazide sharply increases, which indicates that solubility is not a limiting factor for its absorption in this pH range [16].

Given the potential impact of DDI on therapeutic outcomes, it is of special interest to further investigate how simultaneous administration of gliclazide with antacids affects the absorption of gliclazide. Thus, this study aimed to examine the effects of antacids on the permeability of gliclazide using in vitro parallel artificial membrane permeability assay (PAMPA) that simulates absorption in the gastrointestinal tract and to gain a better insight into the mechanisms responsible for the observed effects.

## 2. Results

**Figure 1:**
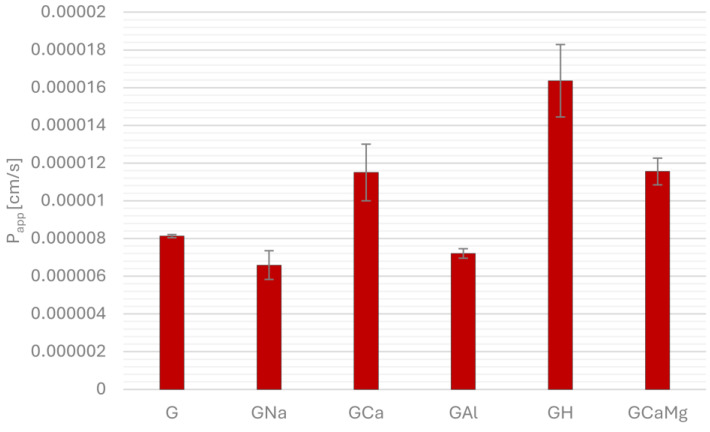
Permeability of gliclazide after six-hour incubation, alone (G) and in combination with sodium bicarbonate (GNa), calcium carbonate (GCa), aluminum hydroxide (GAl), hydrotalcite (GH) and calcium carbonate and magnesium carbonate (GCaMg) at pH 1.2.

**Figure 2:**
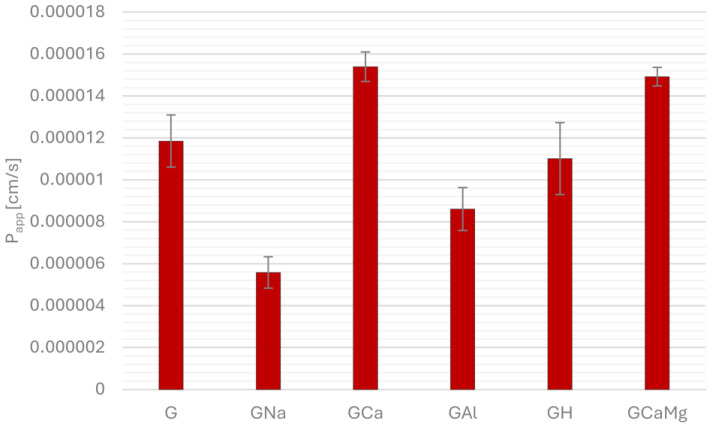
Permeability of gliclazide after six-hour incubation, alone (G) and in combination with sodium bicarbonate (GNa), calcium carbonate (GCa), aluminum hydroxide (GAl), hydrotalcite (GH) and calcium carbonate and magnesium carbonate (GCaMg) at pH 4.5.

**Figure 3:**
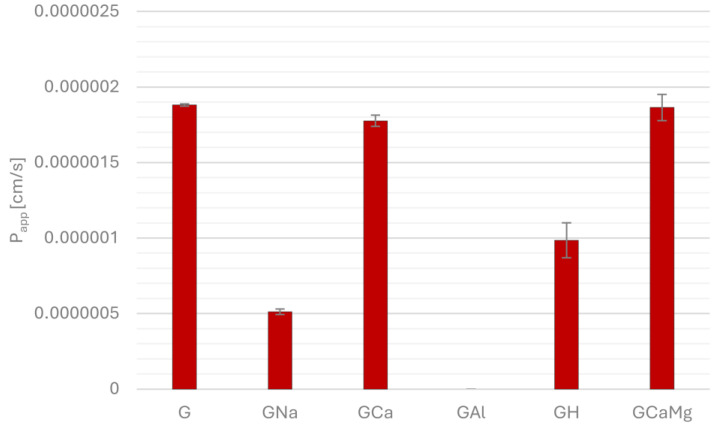
Permeability of gliclazide after six-hour incubation, alone (G) and in combination with sodium bicarbonate (GNa), calcium carbonate (GCa), aluminum hydroxide (GAl), hydrotalcite (GH) and calcium carbonate and magnesium carbonate (GCaMg) at pH 6.8.

**Figure 4:**
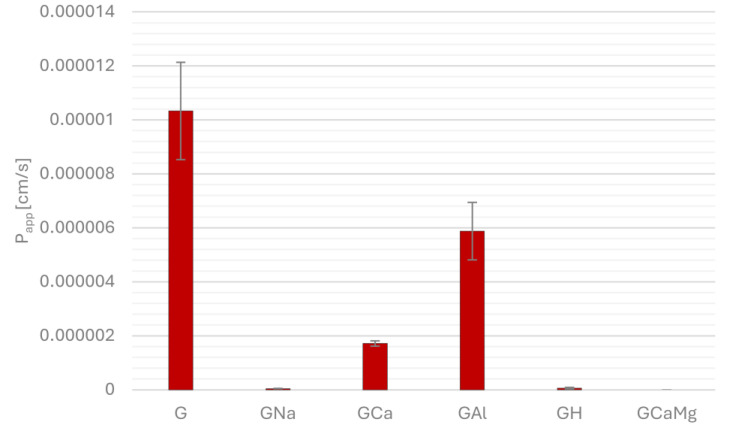
Permeability of gliclazide after six-hour incubation in water, alone (G) and in combination with sodium bicarbonate (GNa), calcium carbonate (GCa), aluminum hydroxide (GAl), hydrotalcite (GH) and calcium carbonate and magnesium carbonate (GCaMg).

Figure 1, Figure 2, Figure 3 and Figure 4 show the permeability of gliclazide in the control groups without antacids (G) and the experimental groups that contained antacids (GNa, GCa, GAl, GH, GCaMg) after a six-hour incubation in buffers at pH 1.2, 4.5, 6.8 and in water. Apparent permeability coefficient (P_app_) was calculated to assess the permeability. Values of the coefficient less than 1.5 × 10^−6^ cm/s indicated poor permeability, whereas higher values suggested good permeability.

At simulated fasting gastric conditions (pH 1.2), after a six-hour incubation, the experimental groups GCa, GH and GCaMg showed statistically significantly better permeability compared to the control group G (G/GCa, GH, GCaMg 8.13 × 10^−6^ ± 8.69 × 10^−8^ vs. 1.15 × 10^−5^ ± 1.50 × 10^−6^, *p* = 0.02366, 1.64 × 10^−5^ ± 1.92 × 10^−6^, *p* = 9.28177 × 10^−6^, 1.16 × 10^−5^ ± 7.07 × 10^−7^, *p* = 0.02123, respectively). Experimental groups GNa and GAl showed poorer permeability than control group G, but without statistical significance (Figure 1).

At fed-state gastric conditions (pH 4.5), groups GCa and GCaMg had significantly better permeability compared to the control group G (G/GCa, GCaMg 1.18 × 10^−5^ ± 1.24 × 10^−6^ vs. 1.54 × 10^−5^ ± 7.04 × 10^−7^, *p* = 0.0149, 1.49 × 10^−5^ ± 4.44 × 10^−7^, *p* = 0.03745, respectively), while groups GNa and GAl had significantly poorer permeability compared to control group G (G/GNa, GAl 1.18 × 10^−5^ ± 1.24 × 10^−6^ vs. 5.58 × 10^−6^ ± 7.53 × 10^−7^, *p* = 1.20 × 10^−4^, 8.61 × 10^−6^ ± 1.02 × 10^−6^, *p* = 0.02685, respectively). Group GH had poorer permeability than control group G, but statistical significance was not reached (Figure 2).

At fasted-state intestinal conditions (pH 6.8), all experimental groups had poorer permeability compared to control group G but with statistically significant differences between G/GNa 1.88 × 10^−6^ ± 7.07 × 10^−9^ vs. 5.12 × 10^−7^ ± 1.84 × 10^−8^, *p* = 0.000, G/GAl 1.88 × 10^−6^ ± 7.07 × 10^−9^ vs. 0, *p* = 0.000 and G/GH 1.88 × 10^−6^ ± 7.07 × 10^−9^ vs. 9.85 × 10^−7^ ±1.16 × 10^−7^, *p* = 2.95 × 10^−6^. Given that in the acceptor compartment of the GAl group, after six hours of incubation, the concentration of gliclazide was below the limit of detection, the permeability coefficient was set to zero (Figure 3).

In water, after a six-hour incubation, it was observed that all groups with antacids GNa, GCa, GAl, GH and GCaMg had significantly poorer permeability compared to the control group G (G/GNa, GCa, GAl, GH, GCaMg 1.03 × 10^−5^ ± 1.8 × 10^−6^ vs. 4.49 × 10^−8^ ± 5.83 × 10^−9^, *p* = 2.53 × 10^−7^, 1.71 × 10^−6^ ± 9.93 × 10^−8^, *p* = 4.28 × 10^−7^, 5.88 × 10^−6^ ±1.07 × 10^−6^, *p* = 3.93 × 10^−4^, 5.89 × 10^−8^ ± 2.67 × 10^−8^, *p* = 2.53 × 10^−7^, 0, *p* = 2.53 × 10^−7^, respectively). In the acceptor compartment of the GCaMg group, after six hours of incubation, the concentration of gliclazide was below the detection limit, and the permeability coefficient was set to zero (Figure 4).

In order to evaluate the pH increase in the medium induced by antacids, the pH of each group was measured, and these values are shown in Table 1.

**Figure 5:**
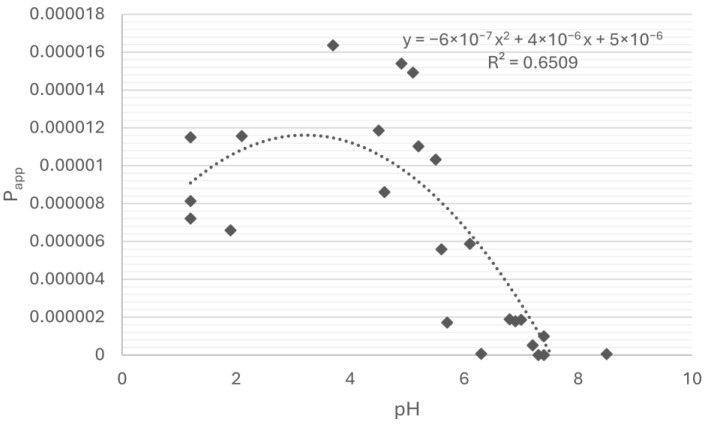
Dependence of gliclazide permeability on pH value.

The dependence of gliclazide’s apparent permeability on the pH value was measured, and their correlation was evaluated. Several regression models were tested, and the best fit was a nonlinear, parabolic relationship described by a quadratic equation (y = −6 × 10^−7^ x^2^ + 4 × 10^−6^ x + 5 × 10^−6^, R^2^ = 0.6509) (Figure 5).

**Figure 6:**
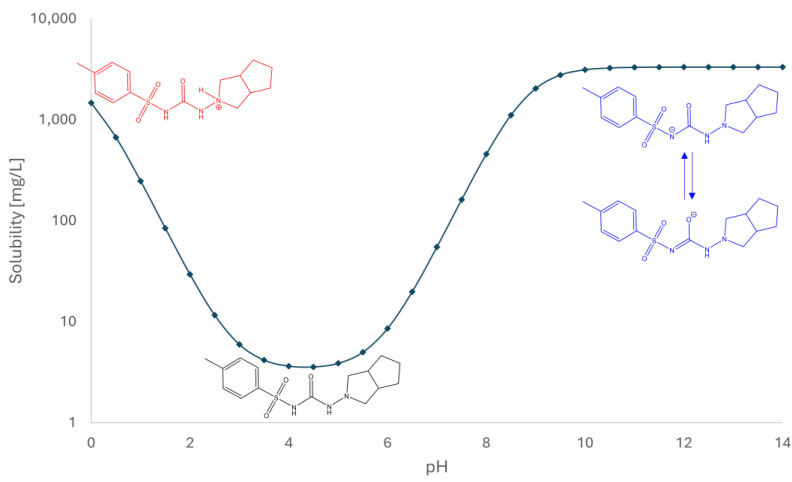
The calculated solubilities of gliclazide in water across the entire pH range (0–14).

PK-Sim solubility modeling demonstrated pH-dependent solubility of gliclazide with a steep increase in solubility from pH 5 to pH 9. The approximate solubility of gliclazide was in the range from 3 mg/L to 3000 mg/L (Figure 6).

## 3. Discussion

DDI refers to the interactions between drugs used in drug therapy, as well as interactions between drugs and diagnostic agents, food, metabolites and endogenous substrates. Thus, they can cause profound clinical effects, resulting in enhanced or reduced therapeutic efficacy, or even enhanced drug toxicity. In general, DDI may be divided into pharmaceutical, pharmacokinetic and pharmacodynamic interactions. Pharmacokinetic interactions occur in the processes of drug absorption, distribution, metabolism and/or elimination when drugs are simultaneously administered [17,18,19]. Polypharmacy drastically increases the risk of DDI and is especially problematic in patients who use OTC drugs without proper medical supervision [20].

Due to the well-known potential of antacids to interact with other medicines, the focus of this research was to investigate their influence on the absorption of gliclazide, which is the antidiabetic drug characterized by a narrow therapeutic range. During concomitant administration, antacids usually reduce or delay the absorption of the co-administered drug. However, they may also lead to an increase in absorption and, consequently, to an increase in the bioavailability of the drug and the risk of side effects, especially in the case of drugs such as oral antihyperglycemics [21,22]. In order to gain insight into the mechanisms of interactions between antacids and gliclazide in the gastrointestinal tract, we examined the permeability of gliclazide across the PAMPA membrane at pH 1.2, pH 4.5, pH 6.8 and in water. PAMPA serves as a rapid, flexible and cost-effective method for estimating trans-barrier permeability of drug substances as well as harmful chemicals. The PAMPA model imitates the actual lipid bilayer of live cells, which makes its application suitable for predicting the passive absorption of orally administered drugs. By careful consideration of experimental conditions, including pH, incubation time and shaking/stirring speed, it is possible to simulate the conditions prevailing in the gastrointestinal environment [23,24]. As previously mentioned, the main mechanism of absorption of gliclazide is passive diffusion, which makes it a suitable candidate for this type of screening.

Given that gliclazide is mainly classified as a weak acid drug with a tendency to ionize at higher pH values, it could be expected that the best permeability is achieved at the lowest pH value [25]. The nitrogen atom of the sulfonylurea group, positioned between the sulfonyl (SO₂) and carbonyl (CO) groups, is weakly acidic and can donate a proton under alkaline conditions (Figure 6). However, gliclazide has amphiphilic properties due to the presence of not only the acidic sulfonamide group (pKa 5.8) but also the basic alicyclic amino group (pKa 2.9). Therefore, it was observed that the permeability of gliclazide in the control group without antacids (G) was better at pH 4.5 than at pH 1.2, since gliclazide is in a minimally ionized state at pH 4.5. While at higher pH values, the sulfonamide group deprotonates and carries a negative charge, at lower pH values (pH 1.2), the alicyclic amino group receives a proton and carries a positive charge. As a result, the highest permeability of gliclazide in the control group was observed at pH 4.5. This result is in accordance with previously published studies [16,26].

Also, by examining the impact of the change in a pH value induced by antacids on the permeability of gliclazide, this study revealed the inverted U-shaped correlation between permeability and pH, which means that there is an optimal pH where the system is most permeable (pH~3–5), and permeability is lower at both acidic and basic extremes. This strength of correlation (R^2^ = 0.6509) supports the hypothesis that ionization may not be the only factor affecting gliclazide permeability and that other mechanisms and factors are likely involved.

Given that the permeability of gliclazide is optimal at pH 4.5, it was anticipated that the presence of antacids would decrease the permeability of gliclazide by affecting the pH in experimental groups where this parameter was maintained at 4.5. This was confirmed in the group treated with sodium bicarbonate, in which the pH value increased, resulting in gliclazide’s significantly lower permeability. However, in the aluminum hydroxide group, although pH 4.5 did not change after the antacid addition, the permeability of gliclazide was significantly lower. This suggests the involvement of another mechanism of interaction, which is most likely the formation of an insoluble chelate complex with aluminum, similar to the interactions observed with ibuprofen, tetracyclines, penicillamine and quinolone antibiotics [9,20,27]. This is additionally supported by the concentration of gliclazide in the acceptor compartment in the group with aluminum (GAl) at pH 6.8, which was below the limit of detection after 6 h. At that pH value, antacids have no effect on the pH, thereby confirming that the statistically significant difference in gliclazide permeability between the aluminum group and the control group can be attributed to the formation of chelate complexes.

Previous studies have shown that many sulfonamide compounds are capable of coordinating to metal ions through the nitrogen and oxygen atoms, forming stable chelates. The coordination of sulfonamide groups with metal ions is influenced by the electronic and steric properties of the ligand, as well as the oxidation state and size of the metal ion. These factors determine the geometry and stability of the resulting complexes [28]. Intriguingly, the interaction of sulfonylurea antidiabetic drugs (e.g., gliclazide) with metal ions has been explored to enhance their pharmacological profiles [29,30]. The magnesium–sulfonamide complex has been associated with enhanced insulin secretion and improved glycemic control in diabetic models [31,32]. While direct evidence of aluminum chelation by sulfonylureas is limited, studies suggest that aluminum can influence the therapeutic activity of these drugs. For example, the administration of aluminum-based complexes with glibenclamide has been shown to modulate oxidative stress and improve insulin sensitivity in diabetic rats [33,34]. The interaction of aluminum with sulfonamides is believed to occur through the oxygen atoms of the sulfonylurea group, similar to calcium and magnesium coordination. However, further research is needed to fully elucidate the binding mechanisms and therapeutic implications of aluminum–sulfonamide complexes.

In our study, at pH 4.5 and pH 1.2, there was a statistically significant increase in the permeability of gliclazide in the groups with calcium carbonate (GCa) and the combination of calcium carbonate and magnesium carbonate (GCaMg). Previous studies have shown that magnesium hydroxide could enhance the rate and extent of absorption of certain sulfonylurea derivatives, such as glipizide and glibenclamide, and consequently alter the blood glucose level [22,27]. Furthermore, studies investigating the influence of magnesium compounds on ibuprofen absorption, which is also a weak acid with pKa 4.4, have suggested that the formation of soluble salts or complexes with ibuprofen could be a potential mechanism underlying the drug’s improved absorption [12]. Although gliclazide exists in the form of three conformational isomers, it was shown that only in the fully extended conformation, which occupies the state with the minimum energy, is it capable of forming a complex with the calcium ion [35]. Moreover, it was demonstrated that the interaction between two molecules of gliclazide and calcium is related to the mode of action of gliclazide in pancreatic cells. These complexes were characterized by IR and H(1)-NMR in a study conducted by Arayne et al. [10].

In the groups with hydrotalcite, at pH 4.5, the permeability of gliclazide was poorer compared to the control without statistical significance, while at pH 1.2, it was statistically significantly better. Hydrotalcite increased the pH from 4.5 to 5 and from 1.2 to 4.5. Apparently, the results of permeability were a direct consequence of the change in the pH. A study in which a hydrotalcite-like matrix was used to improve the release of gliclazide from the formulation showed that intercalation leads to better release but not at all pH values [36]. This difference in pH-dependent drug release is explained by the crystal structure of hydrotalcite itself, which consists of layers with positive ions balanced by interlayers with anions in which gliclazide remained “trapped”. These anions are interchangeable with other inorganic, organic and metal–organic anions or any biologically active molecule that acts as an acid, such as gliclazide [37]. However, this study was conducted with hydrotalcite-like molecules in which CO_3_^2−^ ions were exchanged with NO_3_^−^ ions. This step was very important since it is well known that the CO_3_^2−^ anions in the original hydrotalcite structure are strongly held in the interlayer region, forming double electrostatic bonds, and it is difficult to replace them with other anions using simple ion-exchange procedures. Hence, it is less likely that the commercially available hydrotalcite used in this study could form a complex with gliclazide and thus affect its permeability.

At pH 6.8, it is common for all groups with antacids that the permeability of gliclazide was poorer compared to the control group. However, only groups with aluminum, sodium bicarbonate and hydrotalcite showed statistical significance. Considering the undetectable concentration in the acceptor compartment after 6 h in the group with aluminum, the adsorption, i.e., the formation of an insoluble complex, might be involved. On the other hand, the increase in pH values in the groups containing sodium bicarbonate and hydrotalcite indicates that their influence on the ionization of gliclazide could be the cause of the lower permeability, as in the groups with pH 1.2 and 4.5.

Upon the drug’s oral administration, a multitude of factors come to determine and affect the rate and extent of its absorption from the GIT. These include physicochemical factors of the drug (i.e., the drug’s pKa, lipophilicity, solubility, diffusivity, stability), physiological factors and characteristics related to the dosage form. Indeed, BCS classifies active pharmaceutical ingredients based on their permeability through the GI membrane and the solubility/dissolution in the GI environment, which are established as the two critical determinants of oral drug absorption. The drug’s dissolution in the aqueous medium of the GIT is typically a prerequisite for its oral absorption, implying that inadequate aqueous solubility often hinders the drug’s oral bioavailability and therapeutic efficacy. Therefore, the interplay between solubility and permeability must be carefully balanced in order to optimize the overall oral absorption [38,39,40]. As previously discussed, gliclazide is a BCS class II drug, characterized by low solubility and high permeability. Its absorption is limited by its dissolution rate [41]. The pH-dependent solubility of gliclazide was proposed to be a relevant factor for its slow absorption in the GIT [42]. This is in accordance with the results from PK-Sim solubility modeling conducted in this study, where the pH-dependent solubility of gliclazide aligns well with the measured changes in permeability across different pH levels, with the maximum membrane permeability coinciding with minimal aqueous solubility within the pH range of 3 to 5. It was reported that the range of pH 3.0–4.2 is the main solubility-limited range and that the solubility of gliclazide is the limiting factor for absorption up to pH 6.9 [42]. This suggests that adequate solubility of gliclazide at pH 4.5, as well as the positive influence of calcium and magnesium ions, contributes to its statistically significant increase in permeability.

This study has certain limitations. The interactions between gliclazide and antacids containing different metal ions were not directly confirmed through experimental interaction studies; our conclusions are based on indirect evidence from changes in membrane permeability. Furthermore, the findings are solely based on in vitro PAMPA experiments, which, while useful for assessing passive permeability, may not fully replicate the complexity of in vivo drug absorption. Therefore, further in vivo studies are necessary to confirm the clinical relevance of these observations. The PAMPA method itself is subject to several limitations, as it does not account for the physiological processes of the human upper gastrointestinal tract and microbiota, both of which are crucial for a drug’s in vivo fate. Namely, the acid-neutralizing capacity of antacids to change gastric pH can be easily determined; however, the rate of this reaction depends on gastric secretion and gastric emptying [5]. Also, by altering gastric acidity, antacids may increase the survival rates of food-borne pathogens, i.e., *Vibrio vulnificus* [43]. The reduction in gastric acidity may favor the survival and growth of such pathogens, potentially affecting the overall microbial composition in the gastrointestinal tract. Given the mounting evidence of crosstalk between gut microbiota and gliclazide, any alterations in the composition of gut microbiota may affect the therapeutic efficacy of gliclazide [44,45,46]. Therefore, the interactions between antacids and gliclazide should be investigated using more sophisticated in vitro models that replicate dynamic in vivo conditions and the microbial environment, thereby providing a more expeditious and ethically responsible alternative to traditional animal and human studies. Computational models developed to calculate and evaluate the interaction probabilities between drugs could also offer substantial support to experimental methods [18].

## 4. Materials and Methods

### 4.1. Materials

Gliclazide was obtained from Hemofarm AD, Serbia. The following antacids were used: sodium bicarbonate (Sigma-Aldrich, Darmstadt, Germany), calcium carbonate (Sigma-Aldrich, Germany), aluminum hydroxide (Sigma-Aldrich, Germany) and commercially available antacid preparations Rupurut^®^ (INN: hydrotalcite; Bayer Bitterfeld GmbH, Bitterfeld-Wolfen, Germany) and GastroGuard^®^ (INNs: calcium carbonate, magnesium carbonate; Alkaloid A.D., North Macedonia). Water, acetonitrile and dimethyl sulfoxide (DMSO) were of HPLC grade (J.T. Baker, Phillipsburg, NJ, USA). Hydrochloric buffer pH 1.2, acetate buffer pH 4.5 (0.05 M), phosphate buffer pH 6.8 (0.05 M) and phosphate buffer 7.4 (0.05 M) were prepared following the European Pharmacopoeia guidelines.

### 4.2. Preparation of Solutions

A stock solution of gliclazide (20 mg/mL) was prepared by dissolving it in DMSO. The stock solution was diluted 100-fold with phosphate buffer 7.4 to achieve a final sample concentration of 200 μg/mL. Standard gliclazide solutions for the calibration curve were prepared by diluting the stock gliclazide solution with acetonitrile to the final concentrations in the range of 0.2–50 μg/mL. The dependence of the peak area on the concentration was analyzed. The correlation coefficient of the calibration curve obtained as the dependence of the peak area on the concentration of gliclazide was R^2^ = 1. The calibration curve equation was y = 2.0885x – 0.076.

Considering the typical single doses of gliclazide (60–120 mg) and antacids (300–1000 mg), the antacid concentration in the samples was set to be ten times higher than that of gliclazide (2 mg/mL). All analyses were performed in triplicate in four different media (buffers pH 1.2, pH 4.5, pH 6.8 and water). The pH values of all groups were measured using a pH meter to assess the increase in medium pH caused by antacids.

### 4.3. Permeability Study

To examine the permeability of gliclazide, alone and in combinations with different antacids, a parallel artificial membrane permeability assay (PAMPA) was used. Hydrophobic MultiScreen PVDF microfiltration plates, featuring 96 compartments and a pore diameter of 0.45 μm (Millipore, Billerica, MA, USA), were used as acceptor plates as well as the carriers for the artificial membrane. The acceptor compartments were impregnated with 6 μL of a 2% solution of soy lecithin in n-dodecane, ensuring that the pipette tip did not come into contact with the membrane, and upon the evaporation of the solvent, an artificial membrane was established [47].

The appropriate sections of the acceptor plate were filled with 300 μL of phosphate buffer (pH 7.4), while 300 μL of test compound solutions was added to the donor sections of the MultiScreen Transport Receiver Plate (Millipore, USA). Thereafter, the acceptor plate was gently placed into the donor plate, making sure that the underside of a membrane was in contact with the donor cell solution, without the entrapment of air bubbles. The assembled plates were incubated at room temperature for 2 h and 6 h under continuous slight shaking (50 rpm). After that time, plates were separated, and samples from acceptor compartments underwent HPLC analysis for determination of gliclazide concentration. Samples from the donor compartments were also analyzed after 6 h to check the sum of the gliclazide concentrations in both compartments at the end of incubation time. Samples were diluted 3-fold with water and then injected into the HPLC system.

Following the determination of gliclazide concentration in both compartments, the apparent permeability coefficient (P_app_) expressed in cm/sec was calculated using equations described in our previously published study [25]:

P_app_ = C × [−ln(1 − [C_a_]/[C_eq_])]; (1)

C = (V_d_ × V_a_)/((V_d_ + V_a_) × S × t); (2)

C_eq_ = (C_d_ × V_d_ + C_a_ × V_a_)/(V_d_ + V_a_). (3)

where C_a_ represents the drug concentration in the acceptor compartment, C_eq_ is the drug concentration in equilibrium, V_d_ and V_a_ are the volumes of the solutions in the donor and acceptor compartments (mL), respectively, S is the surface area of the artificial membrane (cm^2^) and t is the permeation time. According to the manufacturer’s data, the area of a filter membrane of the acceptor plate is 0.32 cm^2^, and the porosity is 75%; therefore, the calculated area of an artificial membrane is 0.24 cm^2^.

### 4.4. HPLC Analysis

**Figure 7:**
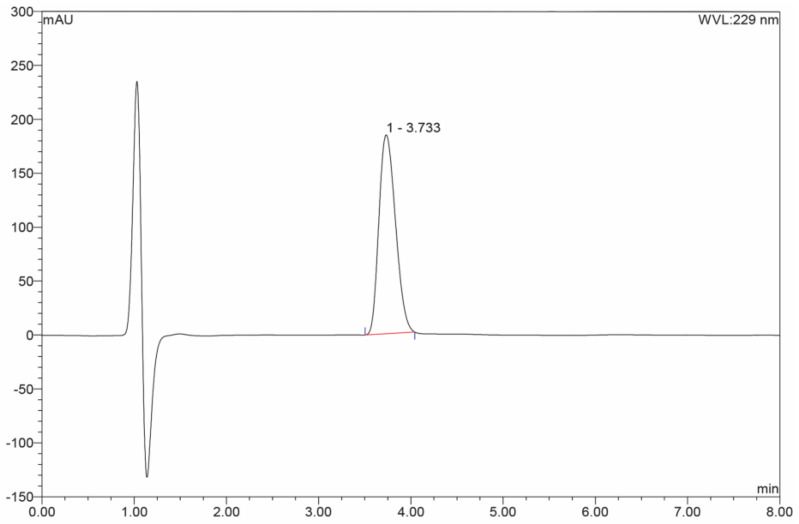
Chromatogram of a sample of gliclazide in donor compartment at pH 6.8.

The concentration of gliclazide was determined by HPLC analysis (Dionex) coupled with a DAD detector using a previously published method [19]. The HPLC analysis was performed using a reverse-phase column Zorbax Eclipse Plus-C18 (100 mm × 2.1 mm, 5 μm, Agilent Technologies, Santa Clara, CA, USA) and guard column Zorbax extend C18 (12.5 mm × 2.1 mm, 5 μm, Agilent Technologies, USA). The injection volume was 20 μL, and the column temperature was 25 °C. The mobile phase for isocratic elution consisted of acetonitrile and water (49:51% *v*/*v*), and pH 2.7 was adjusted by acetic acid, at a flow rate of 400 µL/min. The total run time was 8 min. UV detection was set at 229 nm. The retention time for gliclazide was 3.73 min (Figure 7).

### 4.5. Calculation of pH-Dependent Solubility of Gliclazide in Water

Solubility modeling was performed using PK-Sim 11.2, which is part of the Open Systems Pharmacology Suite (Bayer Technology, Bayer AG, Leverkusen, Germany). The input data are presented in Table 2. The physiochemical properties were taken from the literature data. The solubility values were calculated for gliclazide in a whole pH range of 0–14.

### 4.6. Statistical Analysis

The obtained data were analyzed using statistical software IBM SPSS Statistics, ver. 21 (IBM Institute Inc., Armonk, NY, USA) using one-factor analysis of variance (ANOVA) with Tukey’s post hoc test. To examine the correlation between two numerical variables, we applied several regression models and selected the one with the highest coefficient of determination (R^2^). Statistical hypotheses were tested at the level of statistical significance of 5% (*p* < 0.05) [50].

## 5. Conclusions

The findings of this in vitro study suggest that different antacids can markedly reduce or alternatively enhance the permeability of gliclazide at different pH values. Evidently, this may not only be the result of antacids’ capacity to alter gastrointestinal pH but also the consequence of a direct physicochemical interaction between two drugs. The effect of antacids was of the greatest interest at a pH value of 4.5, since this pH corresponds to the anticipated gastric pH subsequent to the administration of antacids. Additionally, antacids are typically taken 30–60 min after meals, when gastric acid secretion increases during digestion. Gliclazide should be taken with food to minimize the risk of hypoglycemia and promote consistent absorption. Since gliclazide is primarily absorbed in the small intestine, particularly the duodenum and jejunum, the most appropriate choice would be antacids that do not significantly alter its permeability under fed-state gastric or intestinal conditions. In our study, calcium- and magnesium-based antacids met this criterion, since at pH 6.8, which is the pH within the small intestine where the absorption of gliclazide occurs, a decrease in the gliclazide permeability was observed and affected by the presence of sodium bicarbonate, aluminum hydroxide and hydrotalcite. However, while reduced permeability suggests potential bioavailability loss, clinical studies are needed to confirm therapeutic implications.

Given that antacids possess the potential to alter both the rate and extent of absorption of concomitantly administered drugs, future studies should explore the interactions between antacids and different commercially available formulations of gliclazide, particularly those released from dosage forms that are sensitive to pH variations or characterized by pH-dependent stability or solubility. Moreover, taking into account that antacids may alter the absorption of simultaneously administered drugs by affecting intestinal motility, it is crucial to investigate alternative in vitro and in silico models that better simulate physiological processes. Such research may provide a more comprehensive insight into the mechanisms through which antacids impact the absorption and bioavailability of gliclazide and other therapeutics.

Since antacids represent a major class of OTC drugs utilized globally, it is essential that healthcare professionals closely monitor patients who practice polypharmacy, educate them to prevent adverse effects and ensure the therapeutic efficacy of concurrently used drugs.

## Figures and Tables

**Table 1:** Measured pH values in the experimental groups.

Group	G	GNa	GCa	GAl	GH	GCaMg
Buffer pH 1.2						
pH	1.2	1.9	1.2	1.2	3.7	2.1
Buffer pH 4.5						
pH	4.5	5.6	4.9	4.6	5.2	5.1
Buffer pH 6.8						
pH	6.8	7.2	6.9	7.3	7.4	7.0
Water						
pH	5.5	8.5	5.7	6.1	6.3	7.4

**Table 2:** Input data for calculation of pH-dependent solubility of gliclazide in water.

Input Parameter	Value
logP [13]	1.7
Albumin binding [48]	up to 97% (95%)
Unbound fraction [48]	0.05 (5%)
Molecular weight (MW) [13]	323.41 g/mol
Acidic group pKa [13]	5.8
Basic group pKa [13]	2.9
Solubility in water (at 37 °C, pH 7) [49]	55 mg/L

## Data Availability

The raw data supporting the conclusions of this article will be made available by the authors on request.
